# Mitofilin Preservation Mitigates Cardiac Injury in Donation-After-Circulatory-Death Hearts

**DOI:** 10.3390/cells15100920

**Published:** 2026-05-18

**Authors:** Qun Chen, Zachary Kiernan, Gina Labate, Oluwatoyin Akande, Edward J. Lesnefsky, Mohammed Quader

**Affiliations:** 1 Departments of Medicine, Division of Cardiology, Virginia Commonwealth University, Richmond, VA 23298, USA; 2 Division of Cardiothoracic Surgery, Virginia Commonwealth University, Richmond, VA 23298, USA; 3 Department of Cellular, Molecular and Genetic Medicine, Virginia Commonwealth University, Richmond, VA 23298, USA; 4 Richmond Department of Veterans Affairs Medical Center, Richmond, VA 23249, USA

**Keywords:** MPTP, heart transplantation, calpain inhibitor, mitochondria, cyclophilin D, mitofilin

## Abstract

**Highlights:**

**What are the main findings?**

- Identified mitofilin as a substrate of CPN1/2.
- Identified the role of mitofilin in modulating MPTP in DCD hearts.

**What are the implications of the main findings?**

- Administration of a CPN1/2 inhibitor reduces mitochondrial-driven cardiac injury in DCD hearts.
- Administration of a CPN1/2 inhibitor prevents mitofilin degradation in DCD hearts.

**Abstract:**

Donation after circulatory death (DCD) involves unavoidable ischemia–reperfusion injury (IRI). Mitochondrial permeability transition pore (MPTP) opening plays a critical role in DCD heart injury. Activation of ubiquitous calpains, including calpain-1 and calpain-2 (CPN1/2), increases MPTP opening in DCD hearts. Mitofilin, a mitochondrial inner membrane protein that regulates cristae morphology, is also involved in MPTP opening during ischemia–reperfusion. However, it remains unclear whether CPN1/2 activation contributes to mitofilin-mediated IRI in DCD hearts. We first incubated a mitofilin peptide with exogenous CPN1 in vitro to investigate the link between CPN1 activation and mitofilin degradation. Next, we tested whether CPN1/2 inhibition reduces cardiac injury in DCD hearts by preserving mitofilin and limiting MPTP opening. Sprague-Dawley (SD) rat hearts were subjected to 25 min of in vivo ischemia followed by ex vivo perfusion with or without the CPN1/2 inhibitor MDL-28170 (10 µM). In vitro incubation with CPN1 led to mitofilin degradation, confirming mitofilin as a CPN1 substrate. CPN1/2 inhibition significantly reduced infarct size compared with untreated DCD hearts, preserved mitofilin expression, and decreased MPTP opening. These findings indicate that CPN1/2 activation promotes MPTP opening in DCD hearts through mitofilin degradation. Timely inhibition of CPN1/2 represents a promising strategy to reduce cardiac injury and improve DCD heart function.

## 1. Introduction

Heart transplantation is the definitive treatment for end-stage heart failure. Due to the limited availability of donation-after-brain-death (DBD) hearts, donation-after-circulatory-death (DCD) hearts are increasingly used for transplantation [1,2,3,4,5]. There has been a plateau and, more recently, a significant decrease in brain death donors over the past few years [6]. Conversely, DCD organ donation has steadily increased over the past five years, rising from 3% to over 15% of total heart donors. However, more than 70% of DCD hearts remain unutilized due to longer warm ischemia times (>25 min) and the fear of primary graft dysfunction [6].

In addition to ischemia, DCD hearts are also susceptible to reperfusion injury at the time of transplantation, which damages mitochondria and may compromise the suitability of the heart for transplantation [7,8,9,10,11]. Reperfusion injury impairs the electron transport chain and increases susceptibility to mitochondrial permeability transition pore (MPTP) opening [12,13,14,15,16]. Cyclosporin A, an MPTP inhibitor, reduces cardiac injury when applied at reperfusion, highlighting the critical role of MPTP opening in DCD heart injury [13,17]. Mitofilin, a key component of the mitochondrial inner membrane organizing system, maintains cristae structure for oxidative phosphorylation and ATP production [18,19]. Alterations in mitofilin sensitize mitochondria to MPTP opening after ischemia–reperfusion [19,20], likely through its interaction with cyclophilin D via the C-terminal sequence. Mitofilin content decreases following ischemia–reperfusion, suggesting that mitofilin degradation contributes to MPTP sensitization [19,21]. Prior studies demonstrated that the MPTP is more susceptible to opening in DCD hearts [13,22,23,24]; however, the molecular mechanisms underlying this increased susceptibility have not been well described. We hypothesize that degradation of mitofilin plays a key role in modulating MPTP opening associated with DCD-related ischemia. We further propose that activation of calpains contributes to mitofilin degradation in DCD hearts, thereby promoting increased MPTP opening.

Calpain-1 (CPN1) and calpain-2 (CPN2) are ubiquitous Ca^2+^-dependent cysteine proteases present in both the cytosol and mitochondria [25,26,27]. Activation of CPN1/2 increases cardiac injury during ischemia–reperfusion by degrading structural proteins, such as spectrin [28,29,30,31], and impairing mitochondrial function [27]. Inhibition of CPN1/2 with MDL-28170 reduces MPTP opening in isolated rat hearts after ex vivo ischemia–reperfusion [32,33], indicating that CPN1/2 activation sensitizes mitochondria to MPTP opening. During the DCD process, hearts undergo in vivo global ischemia as an obligatory part of the procurement protocol, followed by additional reperfusion injury during transplantation; thus, ischemia–reperfusion is an inherent aspect of DCD transplantation [34]. We previously studied the role of CPN1/2 in DCD rat hearts and found that cleaved spectrin content was increased in DCD hearts compared with continuously beating-heart-donor (CBD) hearts [35]. Increased cleaved spectrin is a biomarker of CPN1/2 activation [31,36,37], supporting the conclusion that CPN1/2 is activated in DCD hearts. In addition, we found that administration of the CPN1/2 inhibitor MDL-28170 decreased cardiac injury in DCD hearts subjected to 35 min of ischemia [38]. Taken together, these findings indicate that CPN1/2 is activated in DCD hearts, resulting in decreased mitochondrial function [35]. However, it remains unclear whether CPN1/2 activation leads to mitofilin degradation and plays a major role in MPTP opening in DCD hearts. In this study, we investigated (1) the link between CPN1/2 activation and mitofilin degradation; (2) whether CPN1/2 activation during the DCD process leads to mitofilin cleavage; and (3) whether administration of a CPN1/2 inhibitor at reperfusion reduces cardiac injury in DCD hearts by preserving mitofilin content and limiting MPTP opening.

## 2. Materials and Methods

The study was conducted in accordance with the Guide for the Care and Use of Laboratory Animals, and the protocol was approved by the Institutional Animal Care and Use Committees of the Richmond VA Medical Center (1651971) and Virginia Commonwealth University (AH00074596) on 9 July 2025. The health and welfare of the animals used in the study were supervised by personnel from both institutions. All experimental animals were cared for in accordance with the ARRIVE guidelines and the Guide for the Care and Use of Laboratory Animals [39]. They were housed in air-conditioned units with a 12 h light/dark cycle and ad libitum access to water and food. They were allowed to acclimatize for one week from the time of arrival at the facility before being used for the study. Every effort was made to minimize animal pain and discomfort throughout the study.

### 2.1. Establishing the Link Between CPN1/2 Activation and Mitofilin Degradation In Vitro

Purified recombinant mitofilin (1 µg/mL; Novus Biologicals, H00010989-Q01, Centennial, CO, USA) was incubated with purified CPN1 (0.002–0.2 U/mL; Sigma-Aldrich, St. Louis, MO, USA) for 30 min at 30 °C in reaction buffer (25 mM Tris-HCl, pH 7.5, 150 mM NaCl, 2 mM DTT, 1.0 mM CaCl_2_, and 0.5% Triton X-100) [40]. MDL-28170 (10 μM) was used as a CPN1 inhibitor [38]. The proteolysis was terminated by the addition of Laemmli sample buffer and heated at 95 °C for 5 min [40]. Immunoblotting was performed to detect mitofilin using a specific antibody (Thermo Fisher Scientific, Waltham, MA, USA; catalog number MA3-940; dilution 1:1000). Samples containing mitofilin alone or mitofilin incubated with CPN1 were subjected to proteomic analysis to identify potential cleavage sites. The samples were first treated with Tandem Mass Tag (TMT) isobaric labels, which react with free protein termini and lysine residues. After labeling, the samples were digested with trypsin and analyzed by LC–MS/MS [41].

### 2.2. Measurement of Cardiac Function and Infarct Size

Male Sprague Dawley (SD) rats (3–4 months old) were divided into three groups: continuously beating-heart-donor (CBD), vehicle-treated DCD (25 min global ischemia), and MDL-treated DCD (25 min global ischemia). Rats were anesthetized with sodium pentobarbital (100 mg/kg, i.p.), placed on a heating pad to maintain core temperature at 37 °C, and intubated with a 14-gauge tube for respiratory support. Cardiac activity was continuously monitored via electrocardiography (EKG). Heparin (300 U, i.p.) was administered and allowed to circulate for 1 min, followed by vecuronium bromide (40 mg/kg, i.m.) as a paralytic [7,42]. In the CBD group, hearts were procured via thoracotomy without in vivo ischemia and perfused on a Langendorff apparatus for 90 min with oxygenated Krebs–Henseleit (K-H) buffer (115 mM NaCl, 4.0 mM KCl, 2.0 mM CaCl_2_, 26 mM NaHCO_3_, 1.1 mM MgSO_4_, 0.9 mM KH_2_PO_4_, 5.5 mM glucose) at 37 °C and constant pressure of 72 mmHg. For DCD groups, ventilator support was withdrawn to induce respiratory arrest and cardiac asystole. After 25 min of ischemia, hearts were procured and perfused with K-H buffer for 90 min. In the MDL-treated group, DCD hearts were perfused with K-H buffer containing MDL-28170 (10 µM). A 10 µM dose of MDL has been shown to decrease cardiac injury in isolated rat hearts following ischemia–reperfusion [43] (Figure 2A). MDL at 10 uM dose has been shown to decrease cardiac injury in isolated rat hearts following ischemia–reperfusion [38]. Our recent study similarly showed that 10 µM MDL decreased cardiac injury in DCD hearts [38]. Thus, MDL (10 uM) was used in the current study to test if inhibition of CPN1/2 can decrease mitofilin degradation in the DCD hearts.

Cardiac function was assessed during reperfusion using a latex balloon-tip catheter inserted into the left ventricle. Heart rate and left ventricular developed pressure (LVDP) were recorded and analyzed with LabChart software (Version 7, ADInstruments Inc., Colorado Springs, CO, USA). The rate-pressure product (RPP = heart rate × LVDP) was calculated to account for heart rate-related variability in cardiac function. Infarct size was determined after 90 min of reperfusion using TTC (2,3,5-Triphenyltetrazolium Chloride) staining [13]. Hearts were frozen at −20 °C for 24 h, then cut into four 2–3 mm slices along the long axis. Slices were incubated in 1% TTC at 37 °C for 20 min and subsequently fixed in 10% formalin at 4 °C for 24 h. After drying and weighing, slices were placed between labeled plastic sheets, scanned, and analyzed with NIH ImageJ (Version 1.54p) to calculate infarct size in a blinded manner [13].

The experimental groups for the assessment of cardiac function and myocardial infarction included: (1) continuously beating-heart-donor (CBD) group (n = 10); (2) vehicle-treated DCD group (n = 9); and (3) MDL-treated DCD group (n = 10). A separate set of animals was used for mitochondrial functional analysis, consisting of CBD (n = 10), DCD (n = 10), and DCD + MDL (n = 9) groups.

### 2.3. Assessing Mitochondrial Function in Isolated Subsarcolemmal Mitochondria (SSM)

SSM were isolated from CBD, untreated DCD, and MDL-treated DCD hearts [21]. The rate of oxidative phosphorylation was assessed in freshly isolated mitochondria using glutamate and succinate as complex I and complex II substrates, respectively.

Calcium retention capacity (CRC) was used to assess the sensitivity of mitochondria to calcium-induced MPTP opening. SSM (400 μg/mL) were incubated in a buffer containing 150 mM sucrose, 50 mM KCl, 2 mM KH_2_PO_4_, 5 mM succinate, and 20 mM Tris/HCl (pH 7.4). MPTP opening was induced by sequential additions of calcium (5 nmol/pulse), and CRC was defined as the total calcium required to trigger MPTP opening. CRC is higher in mitochondria, oxidizing complex II versus complex I substrates [37,44]; hence, succinate was used as a substrate. Extra-mitochondrial Ca^2+^ concentration was measured with 0.5 µM Calcium Green-5N, and fluorescence was recorded at excitation/emission wavelengths of 500/530 nm [37,44].

### 2.4. Western Blotting

Cytosolic or mitochondrial proteins were separated using stain-free 12% or 4–15% Tris-glycine gels (Bio-Rad, Hercules, CA, USA). The total protein content in the gel was digitally analyzed after UV activation using Image Lab 6.0 software (Bio-Rad). Proteins were then transferred from the gel to a PVDF membrane (Fisher Scientific, Hampton, NH, USA) using a semi-dry transfer system (Bio-Rad) for 60 min at 20 V. Blots were blocked for 1 h at room temperature in 5% non-fat dry milk and 1% BSA in TBST, and then incubated overnight at 4 °C with primary antibodies (Table 1) against mitofilin and cyclophilin D (1:1000; Thermo Fisher, PA3-022). After four 5 min TBST washes, membranes were incubated for 1 h with HRP-conjugated secondary antibodies (1:10,000; GE Healthcare, Chicago, IL, USA). Following three 10 min TBST washes, blots were developed using ECL Plus reagents and imaged. Protein signals were analyzed using Image Lab 6.0 [41].

### 2.5. Statistical Analysis

Data are expressed as the mean ± standard error of the mean (SEM). SigmaStat 3.5 (Richmond, CA, USA) was used for statistical analysis. For all analyses, differences between three or more groups were compared using one-way ANOVA, provided the data passed normality and equal variance tests. When a significant F-value was obtained, means were compared using Tukey’s post hoc test for multiple comparisons. Statistical significance was defined as *p* < 0.05.

## 3. Results

### 3.1. Direct Exposure of Mitofilin Peptides to CPN1 Led to Mitofilin Degradation In Vitro

**Figure 1:**
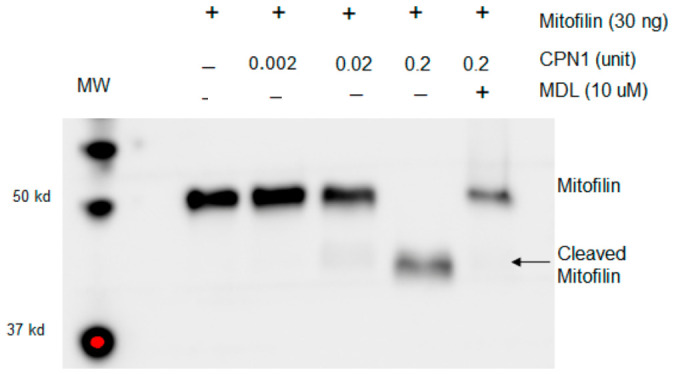
Activation of CPN1 leads to mitofilin cleavage in vitro. Incubation of mitofilin peptides with calpain-1, activated by exogenous calcium, resulted in dose-dependent mitofilin cleavage. Lane 1 contained mitofilin alone, while Lanes 2–4 contained mitofilin with 0.002, 0.02, and 0.2 units of CPN1, respectively. The final lane contained mitofilin with 0.2 units of CPN1 and 10 µM MDL. Inhibition of CPN1 with MDL partially prevented calpain-1–induced cleavage. These results indicate that mitofilin is a substrate of calpain-1.

To investigate the direct interaction between mitofilin and CPN1, the mitofilin peptide was incubated with exogenous CPN1. Immunoblotting revealed that incubation with CPN1 resulted in a dose-dependent decrease in mitofilin content and an increase in cleaved mitofilin (Figure 1). MDL treatment partially prevented CPN1-induced mitofilin cleavage (Figure 1). These results indicate that mitofilin is a substrate of CPN1.

Proteomic analysis identified a total of 10 peptides from the mitofilin sequence, covering 92% of the protein, of which one peptide was labeled with TMT reagents. The TMT-labeled peptide—(20)VQEQELKSEFEQNLSEKLSEQELQFR(45)—had an observed mitofilin/(mitofilin + CPN1) ratio of 1.9. The decrease in this peptide in the CPN1-containing sample suggests N-terminal truncation of mitofilin at the C-terminal side of V20. These results provide further evidence that mitofilin is cleaved by CPN1.

### 3.2. Inhibition of CPN1/2 Decreases Cardiac Injury in DCD Hearts

**Figure 2:**
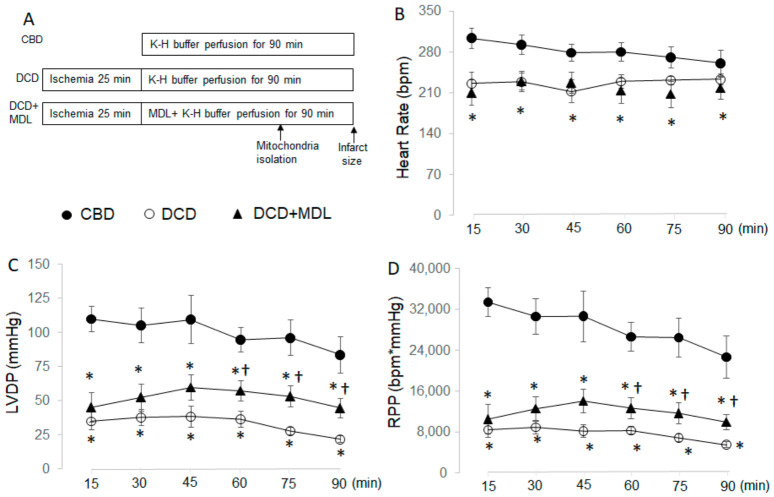
MDL-28170 (MDL) treatment improves cardiac function in DCD hearts. Panel (**A**) shows the experimental protocol, with MDL administered throughout the reperfusion phase. Compared with CBD hearts (closed circles), heart rate (HR) was reduced in DCD hearts (open circles), and MDL treatment (closed triangles) did not improve HR (Panel (**B**)). Left ventricular developed pressure (LVDP) was decreased in DCD hearts compared with CBD throughout reperfusion (Panel (**C**)), and MDL treatment slightly improved LVDP during late reperfusion. Similarly, the rate-pressure product (RPP) was reduced in DCD hearts versus CBD hearts throughout reperfusion (Panel (**D**)), with MDL treatment producing a modest improvement during late reperfusion. Data are expressed as mean ± SEM; * *p* < 0.05 vs. CBD; † *p* < 0.05 vs. untreated DCD hearts. n = 10 in the CBD group, n = 9 in the DCD group, and n = 10 in the DCD + MDL group.

Heart rate (HR) and left ventricular developed pressure (LVDP) were decreased in DCD hearts during reperfusion compared with CBD hearts (Figure 2B,C). The rate-pressure product (RPP, LVDP × HR) was also decreased in DCD hearts compared with CBD hearts (Figure 2D). Left ventricular end-diastolic pressure (LVEDP) was slightly increased in DCD hearts during reperfusion compared with CBD hearts (Table 2). These results indicate that cardiac function was impaired in DCD hearts following ischemia and reperfusion. HR, LVDP, and RPP in MDL-treated hearts remained decreased compared with CBD hearts (Figure 2B–D). MDL treatment did not further increase LVEDP in DCD hearts following reperfusion (Table 2). Although MDL treatment did not improve HR in DCD hearts during reperfusion compared with untreated DCD hearts (Figure 2B), it slightly improved LVDP and RPP, especially during late reperfusion, compared with untreated DCD hearts (Figure 2C,D).

**Figure 3:**
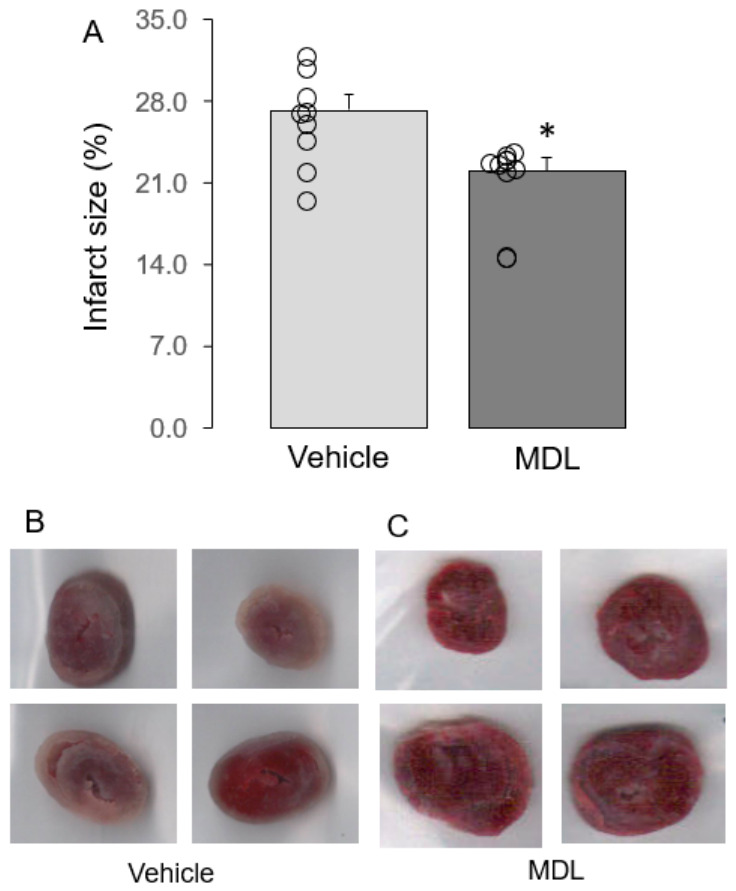
MDL treatment reduces infarct size in DCD hearts. Administration of MDL during reperfusion reduced infarct size compared with untreated DCD hearts (Panel (**A**)). Panels (**B**,**C**) show representative images of infarcted hearts treated with vehicle or MDL. These results demonstrate that CPN1/2 inhibition decreases cardiac injury in DCD hearts. Data are expressed as mean ± SEM; * *p* < 0.05 vs. DCD. n = 9 in the vehicle-treated group and n = 10 in the MDL-treated group.

MDL treatment led to a decreased infarct size in DCD hearts compared to untreated DCD hearts (Figure 3A–C). These results indicate that inhibition of CPN1/2 reduces cardiac injury in DCD rat hearts following ischemia–reperfusion.

### 3.3. MDL Treatment Decreased MPTP Opening in DCD Hearts

**Figure 4:**
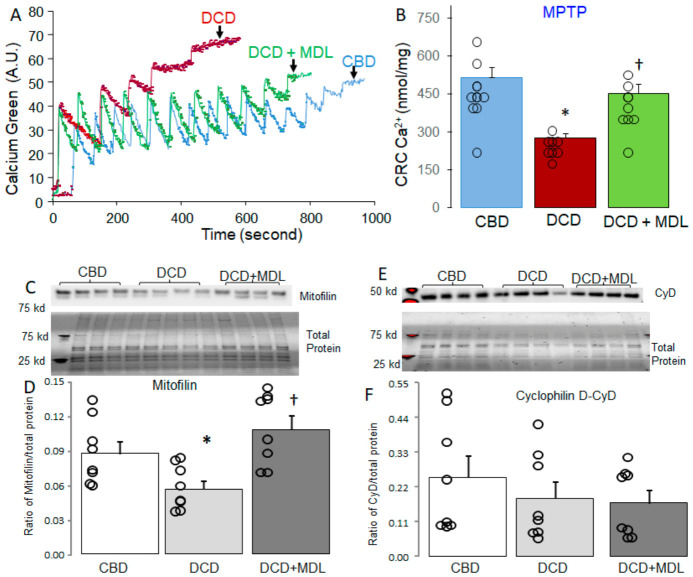
MDL treatment decreases MPTP opening in DCD hearts. Calcium retention capacity (CRC) was used to assess MPTP opening. Panel A shows representative CRC tracings for CBD, DCD, and DCD + MDL groups. CRC was reduced in SSM from DCD hearts compared with CBD (Panels (**A**,**B**)), indicating increased susceptibility to MPTP opening. MDL treatment improved CRC in DCD SSM, suggesting that CPN1/2 inhibition decreases MPTP opening. Mitofilin content was decreased in DCD heart mitochondria compared with CBD (Panels (**C**,**D**)), whereas MDL preserved mitofilin, indicating that mitofilin loss is CPN1/2-dependent. No significant differences in cyclophilin D content were observed among groups (Panels (**E**,**F**)). These findings suggest that mitofilin degradation contributes to increased MPTP opening in DCD heart mitochondria. Data are expressed as mean ± SEM; * *p* < 0.05 vs. CBD; † *p* < 0.05 vs. untreated DCD hearts. For CRC measurement, n = 10 in the CBD group, n = 10 in the DCD group, and n = 9 in the DCD + MDL group. For the Western blotting study, n = 8 per group. (Note: only a subset of samples was used for Western blotting.)

The CRC was used to assess the sensitivity of MPTP opening in isolated SSM. Figure 4A shows the original tracing for the CRC measurement in the CBD, DCD, and DCD + MDL groups. The CRC in SSM isolated from untreated DCD hearts was decreased compared with the corresponding SSM from CBD hearts (Figure 4A,B), suggesting that the sensitivity of MPTP opening is increased in DCD heart mitochondria. MDL treatment increased the CRC in the SSM compared with untreated DCD hearts (Figure 4A,B), indicating that activation of CPN1/2 sensitizes mitochondria to MPTP opening in DCD hearts.

### 3.4. MDL Treatment Decreased the Degradation of Mitofilin in DCD Hearts

Mitofilin content in isolated mitochondria was assessed using immunoblotting. Compared with CBD hearts, mitofilin content was significantly reduced in DCD hearts (Figure 4C,D). MDL treatment preserved mitofilin levels in DCD hearts (Figure 4C,D). These results suggest that the DCD process leads to decreased mitofilin content in a CPN1/2-dependent manner. Cyclophilin D is a key regulator of MPTP opening [45,46,47]. Therefore, cyclophilin D content was measured in CBD and DCD heart mitochondria. There were no significant differences in cyclophilin D levels between the CBD, DCD, and DCD + MDL groups (Figure 4E,F), indicating that the increased MPTP opening in DCD heart mitochondria was not due to changes in cyclophilin D content.

### 3.5. MDL Treatment Decreased the Activation of mCPN1/2 in DCD Hearts

**Figure 5:**
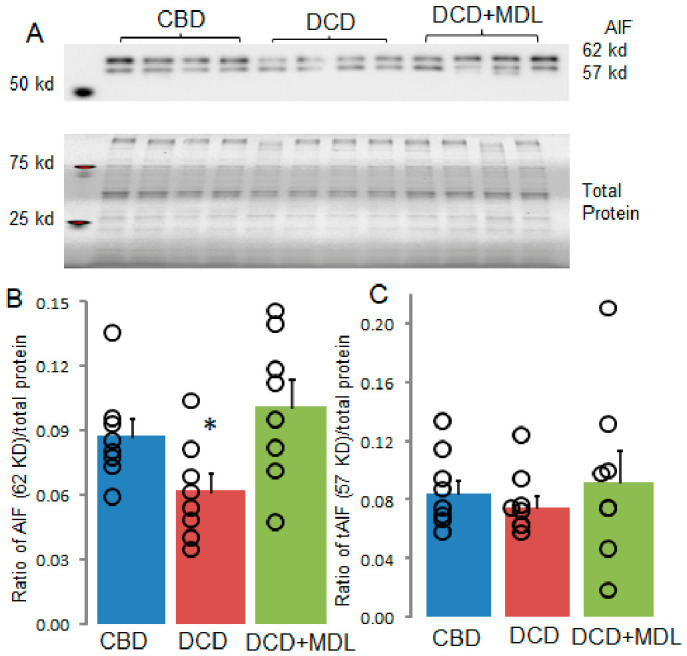
MDL treatment reduces activation of mitochondrial CPN1/2 (mCPN1/2). AIF is a substrate of mitochondrial CPN1/2, and a decrease in AIF content serves as a biomarker of mCPN1/2 activation. AIF content was decreased in DCD hearts compared with CBD (Panels (**A**,**B**)), suggesting activation of mCPN1/2 in DCD mitochondria. MDL treatment preserved AIF content in DCD hearts, indicating that MDL prevents mCPN1/2 activation. No significant differences were observed in truncated AIF (tAIF) content between the CBD, DCD, and DCD + MDL groups (Panels (**A**,**C**)). Although a decrease in AIF would typically result in increased tAIF, the absence of elevated tAIF in DCD mitochondria likely reflects its release into the cytosol. Total protein was used as the loading control. Data are expressed as mean ± SEM; * *p* < 0.05 vs. CBD or DCD + MDL. n = 8 per group. (Note: only a subset of mitochondrial samples was used for Western blotting.)

Our previous study showed that cytosolic CPN1/2 (cCPN1/2) is activated in DCD hearts compared with CBD hearts, and MDL treatment at the onset of reperfusion led to decreased cCPN1/2 activation [35]. In the current study, we tested whether mitochondrial CPN1/2 (mCPN1/2) is also activated in DCD hearts. Apoptosis-inducing factor (AIF) is a known substrate of mCPN1. Activation of mCPN1 cleaves AIF to form truncated AIF (tAIF), which is released from the mitochondria into the cytosol when the permeability of the outer mitochondrial membrane is increased [25]. Mitochondrial CPN2 (mCPN2) may also play a role in AIF cleavage [48]. Thus, a decrease in AIF content within mitochondria serves as a biomarker of mCPN1/2 activation. Compared with CBD hearts, AIF content (62 kDa) was reduced in mitochondria from DCD hearts (Figure 5A,B). However, MDL treatment preserved AIF content in mitochondria from DCD hearts (Figure 5A,B). While a reduction in AIF would typically lead to an increase in truncated AIF (tAIF; 57 kDa) levels in DCD mitochondria, no differences in tAIF content were observed among the groups (Figure 5A,C). Since MPTP opening is increased in DCD heart mitochondria, tAIF may have been released into the cytosol, potentially explaining the absence of elevated tAIF levels in the mitochondrial fraction [48,49]. Our results indicate that mCPN1/2 is activated in DCD hearts and that MDL treatment during reperfusion reduces this activation.

### 3.6. MDL Treatment Did Not Alter Oxidative Phosphorylation in DCD Hearts

The rate of oxidative phosphorylation was assessed in freshly isolated SSM. Compared with CBD hearts, oxidative phosphorylation was significantly reduced in SSM from DCD hearts when glutamate, succinate, and TMPD–ascorbate were used as complex I, II, and IV substrates, respectively (Table 3). These findings are consistent with our previous observations that mitochondrial respiration is impaired in DCD hearts [13]. MDL treatment did not improve oxidative phosphorylation in SSM compared with vehicle treatment in the presence of complex I, II, or IV substrates. Our previous study demonstrated that the reduction in oxidative phosphorylation occurs primarily in DCD hearts subjected to ischemia. In the current study, MDL was administered only during reperfusion, reflecting current clinical DCD practice, in which no interventions are permitted during ischemia. Therefore, it is not surprising that oxidative phosphorylation remained decreased in MDL-treated DCD hearts, as reperfusion-only interventions cannot reverse ischemia-induced mitochondrial damage.

## 4. Discussion

We found that mitofilin is a substrate of CPN1/2 and that CPN1/2 activation promotes mitofilin degradation, leading to MPTP opening in DCD hearts. Inhibition of CPN1/2 at reperfusion using MDL reduced cardiac injury by preserving mitofilin and limiting MPTP opening. Thus, our study identifies a novel mechanism underlying MPTP opening in DCD hearts and provides a clinically relevant strategy to reduce cardiac injury through CPN1/2 inhibition during reperfusion.

Cyclophilin D, a soluble mitochondrial matrix protein, is a key regulator of the MPTP [45,46,47]. Its binding to MPTP components lowers the calcium threshold for pore opening [50,51], and genetic deletion of Cyclophilin D protects MPTP from opening [45]. Pharmacologic inhibition with cyclosporin A during early reperfusion also reduces cardiac injury in DCD hearts [13], highlighting MPTP inhibition as a strategy to protect DCD hearts. In our study, cyclophilin D content was unchanged in DCD hearts, suggesting that increased MPTP opening is not solely due to altered cyclophilin D levels. Recent studies show that mitofilin regulates MPTP opening through interaction with cyclophilin D via its C-terminal sequence [18,20], and mitofilin cleavage dissociates it from Cyclophilin D, promoting MPTP opening [18,19,20]. Our in vitro study showed that incubation of mitofilin peptides with activated CPN1 led to mitofilin cleavage. Inhibition of CPN1 with MDL prevented this cleavage. Proteomic analysis identified potential cleavage sites in mitofilin peptides. Together, these results support mitofilin as a substrate of CPN1/2 and its role in stabilizing the MPTP.

Reperfusion after prolonged ischemia decreases mitofilin content in isolated hearts [19]; similarly, we observed reduced mitofilin levels in DCD hearts compared with CBD hearts. Inhibition of CPN1/2 with MDL preserved mitofilin and decreased MPTP opening. In the present study, we also found that mCPN1/2 is activated in DCD hearts, and that MDL treatment decreases mCPN1/2 activation. Activation of mCPN1/2 augments cardiac injury during reperfusion by impairing mitochondrial function [48]. In the present study, we found that inhibition of mCPN1/2 with MDL protected mitofilin in DCD hearts, further supporting that mitofilin is a substrate of mCPN1/2. Previous studies show that activation of mitochondrial CPN1 increases cardiac injury by enhancing superoxide generation and impairing ATP synthase [52]. Oxidative stress is a key factor that promotes MPTP opening [53,54]. Our study shows that MDL treatment decreased AIF cleavage, indicating reduced mitochondrial CPN1 activation in DCD heart mitochondria. Another study shows that activation of mitochondrial CPN2 sensitizes MPTP opening in rat heart mitochondria by damaging a complex I subunit following ischemia–reperfusion [49]. These findings indicate that MDL treatment may decrease MPTP opening in DCD hearts by reducing ROS generation and attenuating potential complex I damage.

Our previous studies show that the mitochondrial electron transport chain is damaged during ischemia in DCD hearts, as evidenced by decreased oxidative phosphorylation [55]. Eventual improvement in mitochondrial oxidative phosphorylation depends on restoring a healthy mitochondrial population rather than acutely boosting function in already damaged mitochondria [55]. This occurs through mitophagy, a selective form of autophagy that identifies and removes dysfunctional mitochondria via lysosomal degradation [56,57,58]. In parallel, mitochondrial biogenesis, regulated by factors such as PGC-1α, generates new, functional mitochondria to replace those lost [59,60]. Because these quality-control processes require coordinated protein turnover, organelle remodeling, and gene expression changes, recovery of oxidative phosphorylation capacity typically occurs over hours to days rather than during early reperfusion [61]. Thus, it is not surprising that administering MDL during early reperfusion does not improve oxidative phosphorylation in DCD hearts. The mitochondrial permeability transition pore (MPTP) is mainly opened during reperfusion in DCD hearts due to increased oxidative stress or alterations in cyclophilin D status [45,46,47,55]. Administration of cyclosporin A during early reperfusion leads to decreased cardiac injury in DCD hearts [13], indicating that therapeutic intervention during early reperfusion is a feasible approach to reduce cardiac injury in DCD hearts through inhibition of MPTP opening. Our previous studies also show that modulation of ischemia-damaged mitochondria during early reperfusion can decrease MPTP opening in DCD hearts [62]. We found that MDL treatment led to decreased MPTP opening through protection of mitofilin in DCD hearts. These results suggest that MDL-mediated protection occurs mainly through the reduction in MPTP opening during reperfusion in DCD hearts.

In ex vivo evaluations, heart function in CyA-treated DCD hearts did not fully correlate with the reduced infarct size. Both treated and untreated DCD hearts showed lower LVDP compared with CBD controls, although treated hearts exhibited a trend toward improved function during the last 30 min of reperfusion. We attribute this to myocardial stunning, a phenomenon in which reperfused myocardium shows transiently reduced contractility despite adequate perfusion and no overt infarction, as described by Henderickx et al. and observed clinically [63,64]. Theories explaining stunning include oxygen-derived free radical injury and transient calcium overload leading to myofibril dysfunction [65]. While we did not perform magnetic resonance imaging or positron emission tomography to demonstrate stunning, our previous in vivo data showed recovery of heart function in DCD hearts 24 h after transplantation, which was comparable to controls [22]. While myocardial stunning remains a plausible explanation for the lack of functional improvement in CPN1-inhibited DCD hearts, this interpretation is based on our prior studies in DCD hearts subjected to transplantation, where functional recovery was assessed at 48 h [13,22], rather than 90 min following ischemia. In the present model, 90 min of reperfusion on a Langendorff system appears sufficient to detect improvements in MPTP protection and reduction in infarct size; however, meaningful recovery of contractile function following global ischemia may require a longer reperfusion period (e.g., >24 h). This suggests that the early ex vivo functional deficit reflects reversible stunning rather than permanent injury, highlighting an important consideration for clinical decision-making in DCD heart transplantation.

There are several limitations to our study. We acknowledge that multiple simultaneous pathophysiologic processes are in play during cardiac global ischemia and reperfusion-related injury. Our study focused on one of the main mechanisms associated with ischemia–reperfusion injury. Even if we completely protect mitofilin from degradation with MDL, other mechanisms still contribute to ischemia–reperfusion injury in a DCD heart. Since CPN2 is not commercially available, only CPN1 was used in the in vitro studies. Because MDL inhibits both CPN1 and CPN2, the role of CPN2 activation in mitofilin cleavage cannot be excluded. Although we found that mitofilin was degraded in DCD hearts, its specific role in ischemia–reperfusion injury needs to be clarified using genetic approaches, such as knockout or overexpression models, in future studies [20]. Another limitation is the exclusive use of male rats. Our previous work demonstrated that mitochondrial dysfunction and MPTP opening occur to a similar extent in DCD hearts from both sexes [42]. Therefore, we anticipate that CPN1/2 is also activated in female DCD hearts and that its inhibition will similarly reduce cardiac injury. Given the importance of studying drug effects in both sexes, future studies should include equal representation in the study design. Furthermore, MDL-28170 is a non-selective inhibitor of both mitochondrial and cytosolic CPN1/2. It is therefore likely that some of the beneficial effects of MDL treatment are attributable to the inhibition of cytosolic CPN1/2, rather than being specific to mitochondrial calpain-mediated mitofilin degradation. Additionally, post-translational modifications of cyclophilin D, including phosphorylation [66] or acetylation [67] may influence MPTP opening. While cyclophilin D content did not change in DCD hearts, its post-translational modifications may be altered, warranting further investigation. Our findings derived from a small-animal model require confirmation in a large-animal model, such as the pig, to establish translational relevance. Finally, it is possible that the promising results observed in small-animal models may not be reproducible in human hearts.

Our study demonstrates that inhibition of CPN1/2 during reperfusion reduces MPTP opening and cardiac injury in DCD hearts. These findings suggest that timely CPN1/2 inhibition may represent a potential strategy to mitigate ischemia–reperfusion injury and improve the quality of DCD hearts for transplantation, although further validation in clinically relevant models is required.

## Figures and Tables

**Table 1:** Antibodies used in the current manuscript.

Antibody Name	Company	Catalog Number	Concentration
Mitofilin	ThermoFisher Scientific (Waltham, MA, USA)	MA3-940	1:1000
Spectrin	Santa Cruz (Dallas, TX, USA)	csc-46696	1:100
CyD (Cyclophilin 40)	ThermoFisher Scientific (Waltham, MA, USA)	PA3-022	1:1000

**Table 2:** Left ventricle diastolic pressure (LVEDP) in DCD hearts with or without MDL treatment.

Time	CBD (n = 10)	DCD (n = 9)	DCD + MDL (n = 10)
	LVEDP (mmHg)		
15 min	15 ± 2	33 ± 5 *	24 ± 8
30 min	17 ± 2	33 ± 3 *	24 ± 6
45 min	18 ± 2	25 ± 3	29 ± 5
60 min	18 ± 1	26 ± 3 *	28 ± 5
75 min	19 ± 1	32 ± 3 *	31 ± 4 *
90 min	19 ± 1	31 ± 4 *	29 ± 4 *

Mean ± SD. * *p* < 0.05 vs. CBD.

**Table 3:** Rate of oxidative phosphorylation in DCD hearts with or without MDL treatment.

SSM	CBD (n = 10)	DCD (n = 10)	DCD + MDL (n = 6)
	Complex I substrates − Glutamate		
2 mM ADP (nAO/min/mg)	231 ± 76	141 ± 38 *	105 ± 58 *
	Complex II substrate − Succinate + Rotenone		
State 3 (nAO/min/mg)	216 ± 93	142 ± 29 *	148 ± 79 *
	Complex IV substrate − TMPD + ascorbate		
2 mM ADP (nAO/min/mg)	647 ± 267	423 ± 98 *	414 ± 169 *

Mean ± SD. * *p* < 0.05 vs. CBD. Rotenone was used to prevent reverse electron flow when succinate was used as the substrate. TMPD: N,N,N′,N′-Tetramethyl-p-phenylenediamine. Only six mitochondrial samples from the DCD + MDL group were included in the oxidative phosphorylation study.

## Data Availability

The primary results of this research are documented herein and in the Supplementary File S1. Inquiries regarding this work should be addressed to the corresponding author.

## Supplementary Materials

The following supporting information can be downloaded at: https://www.mdpi.com/article/10.3390/cells15100920/s1, File S1: Western Blotting Figure.

## Abbreviations

AIF	Apoptosis-inducing factor
CBD	Control beating-heart donor
CPN1	Calpain-1
CPN2	Calpain-2
CPN1/2	Calpain-1 and calpain-2
DCD	Donation after circulatory death
LVDP	Left ventricular developed pressure
mCPN1/2	Mitochondrial CPN1/2
MDL	MDL-28170
MOPS	3-(N-morpholino)propanesulfonic acid
MPTP	Mitochondrial permeability transition pore
RPP	Rate-pressure product
SD	Sprague Dawley rats
SSM	Subsarcolemmal mitochondria
tAIF	Truncated AIF
TBST	Tris-buffered saline with Tween 20
TMT	Tandem mass tag
TTC	2,3,5-Triphenyltetrazolium Chloride
