# Supplementary material for: Mitofilin Preservation Mitigates Cardiac Injury in Donation-After-Circulatory-Death Hearts
Source: Cells. 2026 May 18;15(10):920. doi: 10.3390/cells15100920 (PMC13204712; doi:10.3390/cells15100920)
Supplement: Supplementary file 1 [file cells-15-00920-s001.zip › cells-4260548-supplementary.pptx]

## Slide 1
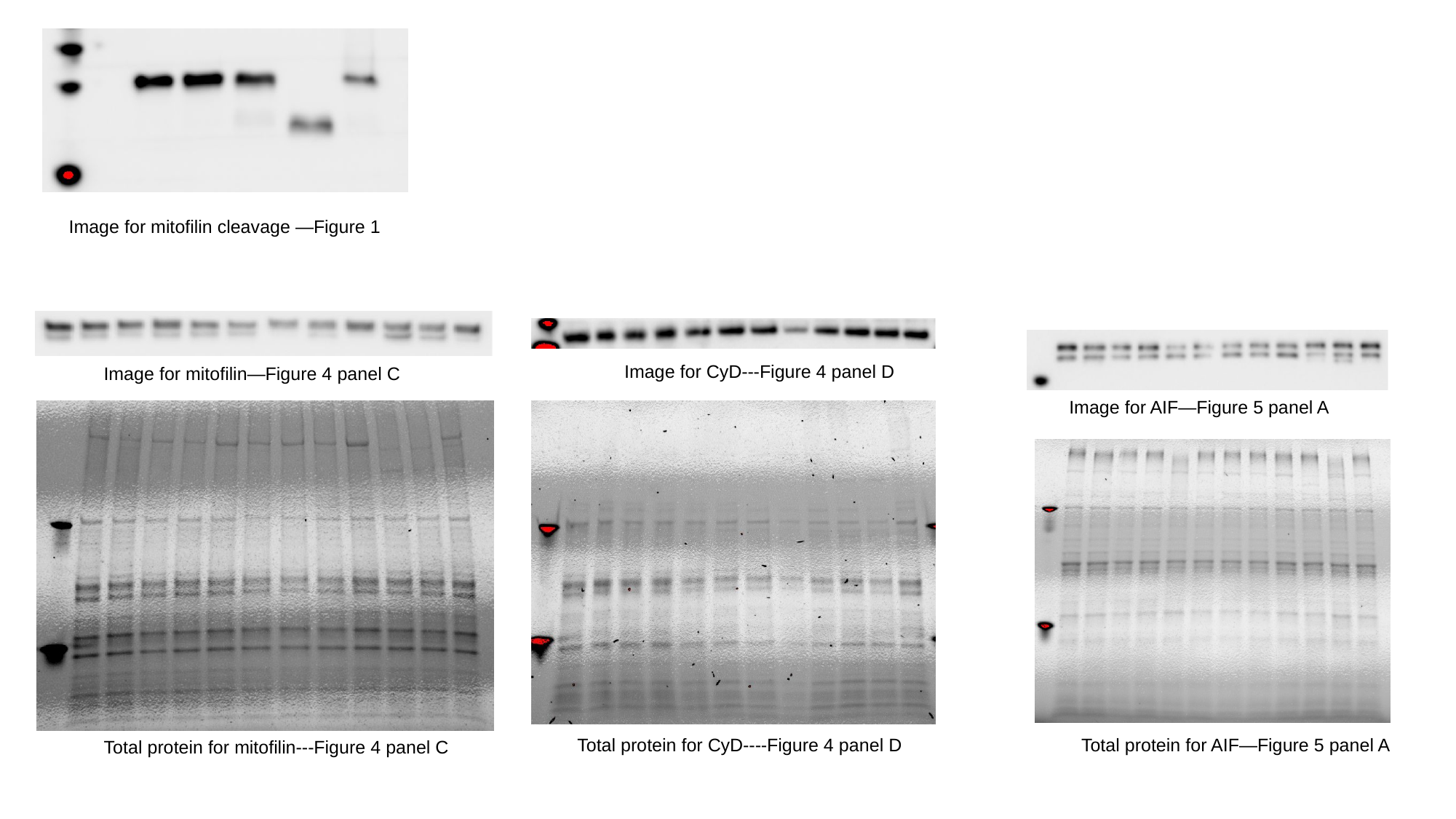

Image for mitofilin cleavage —Figure 1
Image for CyD---Figure 4 panel D
Image for mitofilin—Figure 4 panel C
Image for AIF—Figure 5 panel A
Total protein for CyD----Figure 4 panel D
Total protein for AIF—Figure 5 panel A
Total protein for mitofilin---Figure 4 panel C
